# A Comparative Analysis of the Incidence, Severity and Duration of Smell and Taste Loss in COVID-19 Cases Versus Non-COVID-19 Cases: A Longitudinal Cohort Study

**DOI:** 10.3390/jcm12196267

**Published:** 2023-09-28

**Authors:** Emma J. A. Schepens, Digna M. A. Kamalski, Inge Stegeman, Lifelines Corona Research Initiative

**Affiliations:** 1 Department of Otorhinolaryngology-Head and Neck Surgery, University Medical Center Utrecht, 3584 CX Utrecht, The Netherlands; d.m.a.kamalski@umcutrecht.nl (D.M.A.K.); i.stegeman@umcutrecht.nl (I.S.); 2 Brain Center, University Medical Center Utrecht, 3584 CX Utrecht, The Netherlands

**Keywords:** smell, taste, incidence, severity, course

## Abstract

The COVID-19 pandemic has highlighted the relevance of olfactory and gustatory disorders. However, these symptoms can also be caused by various other factors. In this study we aimed to compare the incidence, severity and duration between COVID-19 related and non-COVID-19 related smell and taste disorders. We conducted a longitudinal cohort study using data from the Dutch biobank Lifelines, which includes over 167,000 participants. The data were collected using 27 questionnaires distributed between March 2020 and May 2022. Descriptive data and the incidence of smell and taste loss in both groups were calculated. To visualize the proportion of severity rates of symptoms, a heatmap was created. A survival analysis was conducted and presented in a reversed Kaplan–Meier curve to show the probability of having persistent smell loss in both groups. The study included 235,722 participants. The incidence of smell loss was higher in the COVID-19 positive group, when compared to the COVID-19 negative group. We found varying degrees of symptom severity in COVID-19 positive cases, ranging from mild to severe, while non-COVID-19 related cases mostly reported mild symptoms. The survival outcome for smell and taste loss was 0.12 (SE 0.03, 95% CI 0.07–0.21) in COVID-19 related cases, and was 0.17 (SE 0.03, 95% CI 0.12–0.24) in cases related to other causes. This study reveals a higher incidence and severity of smell and taste loss in individuals with COVID-19 compared to non-COVID-19 related cases. However, non-COVID-19 related smell and taste loss tend to have a longer duration.

## 1. Introduction

The COVID-19 pandemic has exposed the world to unusual symptoms, namely olfactory and gustatory disorders. The prevalence of non-COVID-19 related smell disorders is reported to be between 1% and 3% [1,2,3]. These causes are mainly sinonasal disorders, post-infectious or post-traumatic disorders, aging and neurodegenerative diseases [1,3,4,5,6,7,8,9]. A recent study revealed that one-third of a general 65-year-old population suffered from impaired smell function and more than one-quarter from impaired taste function [4]. The loss of smell or taste can greatly influence health and overall well-being [5,6]. In COVID-19 cases, smell and taste disorders are among the most common symptoms experienced, which sheds light on the importance of these senses [7,8]. Changes or (partial) loss of the sense of smell or taste have been added to the clinical screening profile for COVID-19 and to the official list of symptoms that persist after COVID-19 by the Centers for Disease Control and Prevention and the World Health Organization [9]. In the majority of individuals with COVID-19, related smell or taste loss recover to normal levels after a few days or weeks [10]. However, it has been established that, 90–150 days after infection, 7.6% of patients still experienced at least a moderate severity of these symptoms (scored on a Likert scale of 1–5) [11]. 

While COVID-19 induced smell and taste disorders are regularly studied, there is a large heterogeneity in these studies, resulting a large variety in their outcomes. This is mainly due to differences in the studied population, reporting systems, healthcare systems, the method of diagnosing COVID-19 and measurement of smell and taste function. Most follow-up data are based on hospitalized COVID-19 patients, which gives a different perspective than information from the general population. Regrettably, data from non-hospitalized patients are not often reported [12]. Moreover, prior to the pandemic, there was limited attention paid to olfactory and gustatory disorders in clinical practice and research [13]. The presence of all of these factors poses challenges when conducting research to compare the course of smell loss between COVID-19 and non-COVID-19 cases.

Therefore, we aim to determine the incidence, severity and duration of COVID-19 induced smell and taste loss, and compare this with non-COVID-19 related smell and taste loss in a general population. 

## 2. Methods

### 2.1. Study Design and Participants 

We conducted a longitudinal cohort study. The data used for this study were collected from a Dutch biobank called Lifelines. Lifelines is a multi-disciplinary prospective population-based cohort study examining in a unique three-generation design the health and health-related behaviors of 167,729 persons living in the north of the Netherlands. It employs a broad range of investigative procedures in assessing the biomedical, socio-demographic, behavioral, physical and psychological factors that contribute to the health and disease of the general population, with a special focus on multi-morbidity and complex genetics. [14,15]. Participants were recruited by general practitioners or online self-registration. Data from participants was assessed by using digital questionnaires. Lifelines uses rigorous protocols for comprehensive data collection to ensure reliability and accuracy of the data [15,16]. The Medical Ethical Committee of University Medical Center Groningen (2007/152) approved the Lifelines cohort study [14,15,16,17]. All Lifelines participants signed informed consent forms [16]. The Lifelines study does not use inclusion criteria; however, severe mental illness, short life expectancy (<5 years), the inability to visit a general practitioner and insufficient understanding of the Dutch language are exclusion criteria [14]. Since April 2020 additional COVID-19 questionnaires have been sent out to Lifelines participants. These participants were at least 18 years old and able to complete digital questionnaires via a valid email address [14,15,17]. Follow-up questionnaires were sent out once a week (questionnaire 1–6) and later on a bi-weekly or monthly basis (questionnaire 7–26) [17]. 

More than 305,500 Lifelines participants were invited to fill in the COVID-19 questionnaires used for this study. Over time, these questionnaires were adjusted; new, relevant topics on COVID-19 were added and some questions were omitted [17].

### 2.2. Procedures

Participants were asked to indicate the extent to which they experienced various symptoms over the past seven days, from March 2020 to May 2020. From May 2020, in order to align with the regular questionnaire distribution schedule (bi-weekly or monthly), the timeframe was adjusted to the past 14 days. Loss of sense of smell or taste was assessed with a 5-point Likert scale (1 = not at all, 5 = extremely). Data were collected from a total of 27 different questionnaires administered between 30 March 2020 and 4 May 2022 (Appendix A). The questionnaires were conducted digitally, and all data, except for age and gender, were self-reported. The definition of a COVID-19 diagnosis has evolved over time. Initially, until 15 May 2020 (questionnaire 7), it was based on a doctor’s diagnosis, due to limited testing options in the Netherlands until August 2020 [18]. From 13 October 2020 to 13 October 2022 (questionnaires 6–14), a diagnosis was established through a doctor’s diagnosis or through a positive PCR test conducted in a healthcare facility. During the period of questionnaire 14 to questionnaire 20 (13 October 2022 to 26 April 2021), only a PCR test conducted in a healthcare facility was considered as a COVID-19 diagnosis, therefore the question about a doctor’s diagnosis was omitted. From 26 April 2021 (questionnaire 20) until 4 May 2022 (questionnaire 26), a positive self-administered home test or a positive PCR test conducted at any organization, such as official testing for events, at work, or at school was considered as a COVID-19 diagnosis. Therefore, individuals who did not receive a doctor’s diagnosis during the specific timeframes when this was required to determine their COVID-19 status, or those who did not undergo testing from questionnaire 14 onwards, were unable to ascertain whether they were positive or negative. Consequently, this led to missing data for these patients, as their COVID-19 status remained unknown. 

### 2.3. Statistical Analysis

All analysis were conducted using SPSS 26.01 and R 4.2.2 statistics software. Data from all 27 different questionnaires were combined and transformed into one dataset. Descriptive statistics were calculated, comparing participants who reported ever experiencing smell loss with participants who never reported experiencing smell loss. The incidence of COVID-19 positive and COVID-19 negative individuals was calculated for every separate questionnaire, each representing a time moment. The incidence is counted as the number or percentage of new cases per moment, and can differ at each time point. 

To visualize the proportion of the severity rates, a heatmap was created using R 4.2.2 statistics software [19,20]. The time for the heatmap is recoded as the moment when the participants first experience smell or taste loss and not based on the moment of completing the questionnaire. Moments 1 to 10 represent the 10 subsequent questionnaires following onset of these symptoms. The reason for including only the 10 subsequent questionnaires after onset of symptoms is due to a high number of participants who had reported no longer experiencing smell loss or due to missing data. To investigate the duration of smell loss in both COVID-19 positive and COVID-19 negative participants, a survival analysis was conducted and visualized by the reversed Kaplan–Meier method using R 4.2.2 statistics software. As well as in the heatmap, only participants who ever reported experiencing smell or taste loss were included in the survival analysis. Having smell or taste loss was defined by a score of 2 or higher on the Likert Scale (1 = not at all, 2 = a little bit, 3 = moderately, 4 = quite a lot, 5 = severely bothered by the symptom). To account for the missing data, participants were combined into two groups (those who ever reported being COVID-19 positive and those who always reported being COVID-19 negative). This grouping was based on all collected data of the 27 questionnaires used for this study, regardless the timing of the participants’ diagnosis (Appendix A). This amalgamation was necessary to compare these two groups in both the heatmap and in the survival analysis. In these analyses, we matched these two groups (COVID-19 positive or negative) to their reported smell loss.

## 3. Results

The overall average response rate for all questionnaires was 36.3%. The average number of responding participants per questionnaire was ~35,000 responders, with the initial questionnaire consisting of ~53,000 responders, who gradually reduced to ~20,000 responders in the final questionnaire [17]. The total number of patients who responded to the COVID-19 questionnaire at least once was 235,722. The median age was 56 years (IQR 49–65). Of the participants, 13,058 (5.5%) reported having smell loss at least once, and 96,300 (40.9%) participants reported never having smell loss. In 126,364 participants (53.6%), this information was missing. Of the patients who ever had smell or taste loss, 6480 participants (49.6%) were female and 3809 (29.2%) were male. There were 4668 (35.8%) participants who were ever diagnosed with COVID-19 and 7110 (54.4%) participants who were never diagnosed with COVID-19; for 1280 patients (9.8%), this information was missing (Table 1).

**Figure 1:**
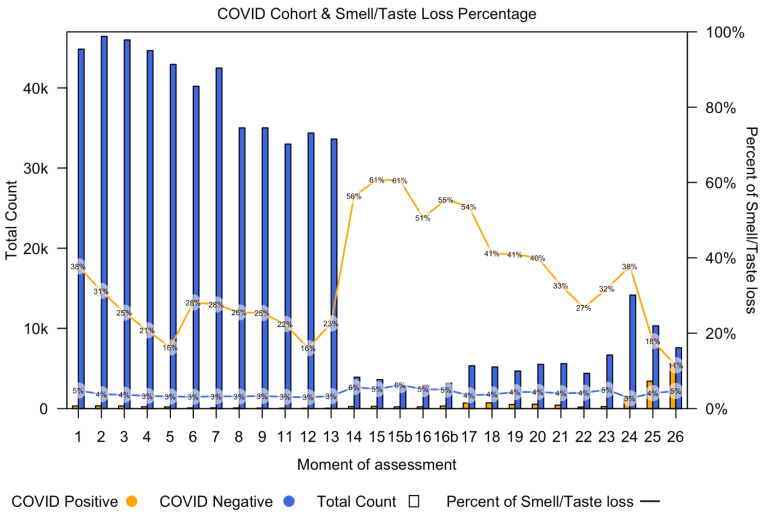
Incidence of participants categorized as COVID-19 positive or COVID-19 negative from first questionnaire (30 March 2020) up to last questionnaire (4 May 2022). The x-axis shows the subsequent questionnaires, each representing a different moment in time. Questionnaire 10 did not contain information about smell and taste or COVID-19 (Appendix A). The orange bars demonstrate the total count of COVID-19 positive participants and the blue bars the total count of COVID-19 negative participants. The orange line demonstrates the percentage of COVID-19 positive participants with smell or taste loss, and the blue line demonstrates the percentage of COVID-19 negative participants with smell or taste loss.

Figure 1 shows the number of COVID-19 positive participants (orange bars) and the number of COVID-19 negative participants (blue bars) per time point, starting at questionnaire 1 (30 March 2020) and going up to questionnaire 26 (4 May 2022). Figure 1 also demonstrates the percentage of participants with smell or taste loss in both the COVID-19 positive group (orange line) and COVID-19 negative group (blue line) for each questionnaire.

From timepoint 14, the number of participants decreased, which is attributed to changes in the criteria for considering a COVID-19 positive or negative status. A doctor’s diagnosis was omitted from the questionnaire and only patients with a positive PCR test were included. As a result, patients without a positive PCR test were considered as missing data, leading to a reduced number of participants classified as either having or not having COVID-19. From questionnaire 20 onwards, self-administered home tests were added into the questionnaires. All participants, regardless of being tested for COVID-19, filled in the questions about smell or taste loss. This could be the reason for the increased incidence of smell loss in COVID-19 cases at timepoint 14.

As shown in the figure, the total number of COVID-19 negative participants is higher than the number of COVID-19 positive participants at every time point. The incidence of smell loss was higher in the COVID-19 positive group, as a consistently higher percentage of COVID-19 diagnosed participants were experiencing smell loss at all time points.

**Figure 2:**
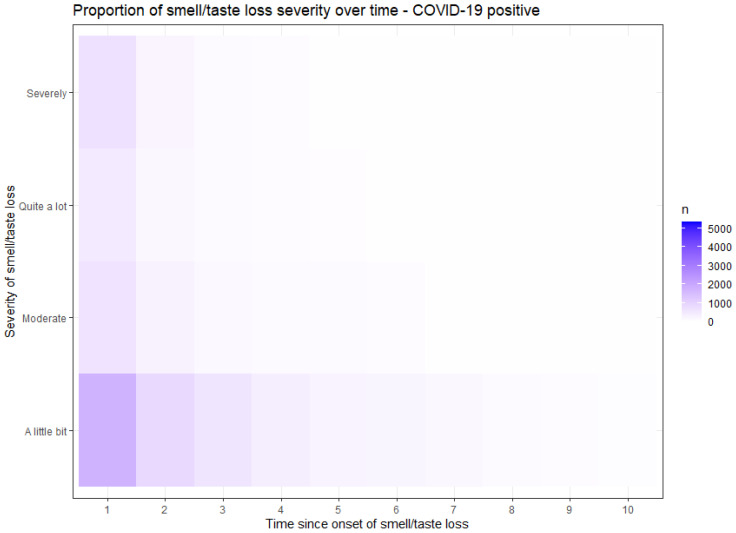
Proportion of severity of smell or taste loss in COVID-19 positive participants, presented in a heatmap. The y-axis shows the rate of severity of smell loss. The time on the x-axis is recoded and not based on the moment of questionnaire assessment, but on the onset of smell or taste loss and subsequent questionnaires with reported ongoing symptoms.

**Figure 3:**
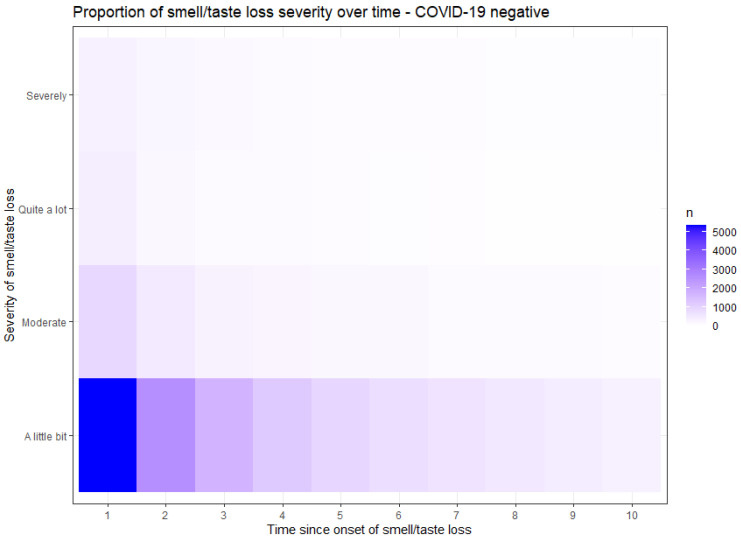
Proportion of severity of smell or taste loss in COVID-19 negative participants, presented in a heatmap. The y-axis shows the rate of severity of smell loss. The time on the x-axis is recoded and not based on the moment of questionnaire assessment, but on the onset of smell or taste loss and subsequent questionnaires with reported ongoing symptoms.

Figure 2 and Figure 3 demonstrate the proportion of the severity of smell or taste loss within the COVID-19 positive (Figure 2) and the COVID-19 negative (Figure 3) group. The y-axis presents the rate of the severity. The x-axis time values are recoded; moment 1 represents the point when participants initially experienced a loss of smell or taste, irrespective of the questionnaire completion moment. The subsequent time points correspond to subsequent questionnaires in which the participants reported their ongoing loss of smell. In COVID-19 related smell or taste loss, the severity was widely distributed from ‘a little bit’ to ‘severely’ (Figure 2). In non-COVID-19 related smell or taste loss, the severity was primarily reported as ‘a little bit’ (Figure 3). These findings show that participants with COVID-19 related smell or taste loss experienced a higher severity than participants with other causes of these symptoms.

**Figure 4:**
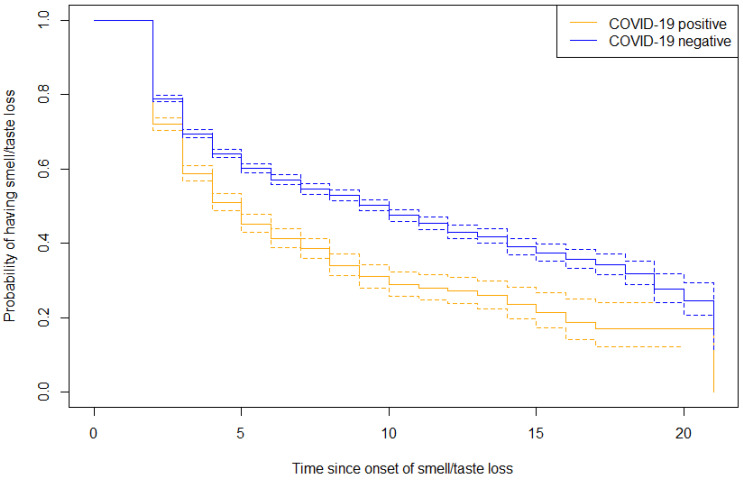
Survival analysis expressed in a reversed Kaplan–Meier curve. The y-axis shows the probability rate of having smell or taste loss. The time on the x-axis is recoded and not based on the moment of questionnaire assessment, but on the onset of smell or taste loss and subsequent questionnaires with reported ongoing symptoms.

The duration of smell or taste loss in both groups was analyzed in a survival analyses and presented in a reversed Kaplan–Meier curve (Figure 4). At the latest measured time point with ongoing symptoms, the survival outcome for COVID-19 related smell or taste loss was 0.12 (SE 0.03, 95% CI 0.07–0.21). The survival outcome for non-COVID-19 related smell or taste loss was 0.17 (SE 0.03, 95% CI 0.12–0.24). These findings suggest that non-COVID-19 related cases exhibit a more prolonged duration, compared to those associated with COVID-19.

## 4. Discussion

There are three main findings of this study. Firstly, we found a higher incidence of smell or taste loss among participants diagnosed with COVID-19, compared to those without COVID-19. It is noteworthy that the majority of participants, whether COVID-19 positive or negative, did not experience these symptoms. Secondly, we observed that participants with COVID-19 related smell or taste loss experienced more severe symptoms, when compared to non-COVID-19 related cases. This could be attributed to the fast and complete onset of olfactory loss in COVID-19, resulting in significant challenges in daily life [21,22]. Thirdly, the duration of smell or taste loss demonstrated a more favorable outcome in COVID-19 participants, as non-COVID-19 related cases were estimated to have a higher likelihood of prolonged symptoms. Consequently, individuals with smell or taste loss originating from other causes than COVID-19 may be prone to extended symptoms or potentially no complete recovery. This is not surprising, since common causes of non-COVID-19 related smell loss are mostly chronic conditions such as sinonasal disorders, post-infectious or post-traumatic disorders, aging or neurodegenerative diseases [1,3,4,13,23,24,25].

The nature of this study, that relied on questionnaire data collected at various time points, comes with certain methodological limitations. The first is missing data, which resulted in temporal gaps between the presented time points. In the heatmap and survival analysis, we therefore modified the time variable to subsequent completed data since onset of symptoms.

Furthermore, it is important to acknowledge the possibility that participants may be COVID-19 positive but experience non-COVID-19 related conditions such as rhinosinusitis or allergies. In such situations, participants were allocated to the COVID-19 positive group. Alongside the fact that there were variations in criteria used for determining COVID-19 positivity or negativity throughout the study period, these factors may have led to either an underestimation or overestimation of the true underlying cause of smell and taste loss.

Despite the inherent challenges associated with data collection through questionnaires, the Lifelines cohort consists of a large and diverse sample from a general population, including multiple repeated measurements, making it highly representative of the general population in the Netherlands. Especially in comparison to other studies where only the relevant concerned participants were included [26,27,28,29], this analysis incorporated a broad group of individuals, making it possible to compare COVID-19 positive cases with COVID-19 negative cases.

Remarkably, a large group of people with non-COVID-19 related smell or taste loss was discovered in this study. These patients have been under the radar for a long time. Before the pandemic, no questions regarding smell or taste were included in any Dutch cohort [14,15,16,17,30,31,32,33,34,35]. Thanks to the impact of COVID-19, the senses of smell and taste have now gained attention, as well as the unfortunate consequences associated with their loss. It is important to use this momentum to focus on patients suffering from this invalidating loss, no matter the cause.

## 5. Conclusions

The incidence of smell or taste loss is higher and more severe when induced by COVID-19 in comparison to non-COVID-19 related smell or taste loss, but the duration is longer in non-COVID-19 related causes.

## Figures and Tables

**Table 1:** Descriptive data. Data are presented as median (IQR) or as *n* (%).

	Ever Experienced Smell/Taste Loss n = 13,058	Never Experienced Smell/Taste Loss n = 96,300
Age, years	56 (48–64)	57 (49–65)
Female Male Missing	6480 (49.6%) 3809 (29.2%) 2769 (21.2%)	46,769 (48.5%) 29,740 (30.9%) 19,791 (20.6%)
Ever had COVID-19 Never had COVID-19 Missing	4668 (35.8%) 7110 (54.4%) 1280 (9.8%)	8045 (8.4%) 54,122 (56.2%) 34,133 (35.4%)
Hospitalized Not hospitalized Missing	158 (1.2%) 4598 (35.2%) 8302 (63.6%)	104 (0.1%) 8880 (9.2%) 87,406 (90.7%)

## Data Availability

Data may be obtained from a third party and are not publicly available.

## Supplementary Materials

The following supporting information can be downloaded at: https://www.mdpi.com/article/10.3390/jcm12196267/s1.

## Appendix A

Table A1 shows the 27 COVID-19 questionnaires (COVQ) used for this study, with the time period of assessment and their response rates [17]. Questionnaire 10 did not contain questions about smell/taste or COVID-19 and is therefore not included.

**Table A1:** The 27 COVID-19 questionnaires (COVQ) used for this study.

COVQ	Date	Response	Response Rate
1	30 March to 23 April 2020	~53,000	41%
2	2 April to 6 May 2020	~51,000	39%
3	12 April to 6 May 2020	~50,000	38%
4	16 April to 13 May 2020	~47,000	36%
5	19 April to 20 May 2020	~45,000	35%
6	28 April to 27 May 2020	~43,000	33%
7	15 May to 29 May 2020	~45,000	34%
8	23 May to 24 June 2020	~38,000	34%
9	11 June to 29 June 2020	~37,000	33%
10	7 July to 29 July 2020	~33,000	30%
11	10 July to 5 August 2020	~35,000	32%
12	24 July to 2 September 2020	~36,000	32%
13	8 September to 30 September 2020	~35,000	32%
14	13 October to 4 November 2020	~34,000	31%
15	2 November to 26 November 2020	~34,000	31%
15 b	15 November to 10 December 2020	~33,000	30%
16	2 December to 21 December 2020	~31,000	28%
16 b	8 December to 5 January 2021	~32,000	29%
17	5 January to 8 February2021	~34,000	31%
18	25 February to 25 March 2021	~32,000	49%
19	29 March to 22 April 2021	~29,000	45%
20	26 April to 20 May 2021	~29,000	44%
21	25 May to 18 June 2021	~29,000	45%
22	4 July to 30 July 2021	~24,000	36%
23	11 October to 4 November2021	~23,000	37%
24	20 December 2021 to 12 January 2022	~25,000	48%
25	28 February to 24 March 2022	~20,000	38%
26	11 April to 4 May 2022	~20,000	40%
