# Supplementary material for: A Comparative Analysis of the Incidence, Severity and Duration of Smell and Taste Loss in COVID-19 Cases Versus Non-COVID-19 Cases: A Longitudinal Cohort Study
Source: J Clin Med. 2023 Sep 28;12(19):6267. doi: 10.3390/jcm12196267 (PMC10573822; doi:10.3390/jcm12196267)
Supplement: Supplementary file 1 [file jcm-12-06267-s001.zip › jcm-2597309-supplementary.pdf]

Lifelines Corona Research Initiative:

H. Marike Boezen [3], Jochen O. Mierau [4,5,6], H. Lude Franke [7], Jackie Dekens [7,8], Patrick Deelen [7], Pauline Lanting [7], Judith M. Vonk [3], Ilja Nolte [3], Anil P. S. Ori [7,9], Annique Claringbould [7], Floranne Boulogne [7], Marjolein X. L. Dijkema [7], Henry H. Wiersma [7], Robert Warmerdam [7], Soesma A. Jankipersadsing [7], Irene van Blokland [7,10] Geertruida H. de Bock [3], Judith GM Rosmalen [9,11] and Cisca Wijmenga [7]

[3] Department of Epidemiology, University Medical Center Groningen, University of Groningen 9713 GZ Groningen, The Netherlands
[4] Department of Economics, Econometrics & Finance, Faculty of Economics and Business, University of Groningen, 9712 CP Groningen, The Netherlands
[5] Lifelines Cohort Study and Biobank, 9300 AB Groningen, The Netherlands
[6] Team Strategy &External Relations, University Medical Center Groningen, University of Groningen,
9713 GZ Groningen, The Netherlands
[7] Department of Genetics, University Medical Center Groningen, University of Groningen 9713 GZ Groningen, The Netherlands
[8] Center of Development and Innovation, University Medical Center Groningen, University of Groningen, 9713 GZ Groningen, The Netherlands
[9] Department of Psychiatry, University Medical Center Groningen, University of Groningen 9713 GZ Groningen, The Netherlands
[10] Department of Cardiology, University Medical Center Groningen, University of Groningen, 9713 GZ Groningen, The Netherlands
[11] Department of Internal Medicine, University Medical Center Groningen, University of Groningen 9713 GZ Groningen, The Netherlands
